# How does ego-depletion affect the reemployment intention of county public sector retirees? The mediating effect of psychological ownership and the moderating effect of social trust

**DOI:** 10.3389/fpsyg.2025.1657287

**Published:** 2025-10-15

**Authors:** Xueqi Zhang, Fengjuan Yan, Lin Meng

**Affiliations:** ^1^School of Labor Relations, Shandong Management University, Jinan, China; ^2^School of Public Administration, Shandong Normal University, Jinan, China

**Keywords:** reemployment intention, ego-depletion, psychological ownership, social trust, public sector retirees

## Abstract

In recent years, with the increasingly severe situation of population aging in China, it had become one of the important ways to implement the active aging strategy and promote the reemployment of the retired young healthy elderly people. At present, there were quite a number of young public sector workers in China who were still able to work at the county level and were forced to quit their jobs due to retirement. To promote the reemployment of these precious labor resources was of great significance to alleviate the problem of labor shortage in China. In this paper, ego-depletion was divided into three dimensions: cognitive depletion, emotional depletion and behavioral depletion. The influence of various dimensions of ego-depletion on reemployment intention was systematically analyzed. The effect of psychological ownership and social trust in the relationship between ego-depletion and reemployment intention was analyzed. The results showed that: (1) All dimensions of ego-depletion had a negative effect on the reemployment intention of younger public sector retirees. The effects of each dimension were as follows: cognitive depletion > behavioral depletion > emotional depletion; (2) Cognitive depletion and emotional depletion had significant negative effects on psychological ownership; Behavior depletion had no significant effect on psychological ownership. (3) Psychological ownership played a mediating effect in the inhibition of reemployment intention by cognitive depletion and emotional depletion; There was no effect on the effect of behavioral depletion on reemployment intention; (4) Social trust positively moderated the relationship between ego-depletion and psychological ownership; In addition, social trust positively moderated the effects of cognitive depletion and behavioral depletion on reemployment intention, but had no moderating effect on the relationship between emotional depletion and reemployment intention.

## 1 Introduction

In recent years, the proportion of elderly people in China has been increasing. By the end of 2023, China had more than 297 million people aged 60 or above, accounting for 21 percent of the country’s population, according to official data. Among them, the younger elderly aged 60 to 69 account for more than half of the elderly aged 60 and above, which is far beyond the international standard for an aging society. According to a survey report on reemployment of the Elderly after Retirement in 2022 released by 51Job.com, an online recruitment platform, China’s younger elderly (aged 60–69) have a strong intention to reemployment. Most of the young elderly have good physical condition, rich knowledge, experience, skills and deep social network, and have important human resource value. Therefore, in the context of active aging, promoting the reemployment of the retired young healthy elderly is one of the important ways to cope with the aging population in China, and also an important way to alleviate the problem of labor shortage. According to the Communique on the Development of the National Cause for Aging in 2022, by the end of 2023, the number of employees participating in the basic pension insurance in China was 370.94 million people. Among them, the number of retired people was 136.44 million, and the number of retired people in government agencies and public institutions was about 16 million. Official data show that the number of elderly people aged 65 and above in China will reach 310 million in 2035 and close to 380 million in 2050. The proportion of the total population reached 22.3 and 27.9%, respectively, and the degree of aging in China will become increasingly severe.

Population aging is the core factor affecting social progress, economic development, spiritual civilization, national comprehensive strength and international competitiveness. It is urgent for the whole country to open up a path with Chinese characteristics to actively cope with the aging population. Studies have shown that a considerable number of retirees who are still able to work in China have been forced to quit their jobs. Some of these retirees have a fairly high level of human capital after years of accumulation of knowledge, skills and experience (Hanif et al., 2018). But at present this precious resource is not being used properly (Xu et al., 2023). The young retirees in this paper refer to the old people who have retired for less than 10 years, which are characterized by good health, clear mind, self-care and strong desire for reemployment. As a treasure house of the wisdom and strength of the aged, the younger public sector retirees are an important part of the aged group and a relatively special group. They include both civil servants and establishment personnel, and are an important force in promoting social stability and development. As a valuable human resource, the younger public sector retirees have deep personal experience, great social influence, much practical experience, strong personality charm, and generally have the value concept and internal consciousness of transcending self-interests and serving the people. They still have enthusiasm, strength and responsibility for their work, and they are precious human resources that are rare for social development. We should encourage them to actively participate in economic, political, social, cultural and other social activities, and play an effect in the process of participation and realize value.

Among them, retired county public sector personnel used to be the indispensable cornerstone of social stability and public services, directly facing the masses and serving the masses. They have directly made important contributions in various fields such as education, health care, social security and environmental protection. Moreover, with the gradual increase in the number of township public sector personnel recruitment in recent years, the county has a huge retired public sector personnel resources every year. Therefore, the overall number of retirees in the county public sector is sufficient and the human resources are abundant. In addition, due to the complexity and change of grass-roots affairs, county retired public sector personnel have rich theoretical knowledge and practical experience, have strong human resources coordination ability, rich experience in social participation, outstanding professional quality, ideological and political integrity, and have reemployment advantages that ordinary elderly groups lack. They should be participants in social governance, and they should also be participants in social outcomes. Therefore, in the era of deepening aging, it is crucial to continue to give full play to the unique advantages of younger public sector retirees in counties to solve the problem of aging and alleviate the shortage of labor resources in China.

At present, the academic research on the factors affecting the retirement and reemployment of county public sector personnel can be roughly divided into two aspects: on the one hand, the micro level. The researchers mainly analyzed the factors affecting the reemployment of public sector employees after retirement from the perspective of individuals and families. At the individual level, a number of studies have shown that gender, age (Anser et al., 2020), education level (Cai and Kosaka, 2019), physical health status (Alraddadi, 2021), personal intention to work (Thomassen et al., 2020) and professional skills (Ma et al., 2022) have a significant effect on the reemployment of public sector retirees. Some scholars have pointed out that in the case of the same factors, the level of education plays a more important and positive effect, and the concept of reemployment also has an important effect on the actual employment situation (Underwood et al., 2020). However, other studies have found that the effect of education level on reemployment is not significant (Kocak et al., 2023). At the family level, some studies have pointed out that family support has a significant effect on the reemployment intention of the young retired elderly in urban areas (Shamsikhani et al., 2021). The marital status (Zhang et al., 2024), number of children (Hapunda, 2023), attitude of children and inter-generational support (Zhao et al., 2023) of retirees in their later years have an important effect on reemployment. Some researchers believe that retirees’ marital status, the number of children, and children’s economic support have no significant effect on reemployment (Zhang et al., 2022). It is found that pension level (Zhao et al., 2019), social security system, salary system, employment policy (Cohen, 2022) and labor market mechanism (Andersen and Sundstrup, 2019) all have a significant effect on retirement and reemployment intention. With the progress of the society, the influence of the Internet (Li and Wang, 2023) has also attracted the attention of some scholars.

As an important individual cognitive factor, psychological ownership describes a sense of possession and has an important effect on the choice of behavior (Yu and Wang, 2022). Studies have shown that psychological ownership arises from individual’s social identity, sense of efficacy and self-identity, which can awaken individual’s sense of social responsibility and promote individual’s active participation in organizational construction (Abubakar et al., 2017). Ego-depletion theory points out that an individual’s self-control resources are limited in a certain period of time (Wrede et al., 2021). Negative life experiences or stress and previous self-control demands and so on deplete self-control resources. The state of depletion of self-control resources is ego-depletion. When the individual is in a state of ego-depletion, it will cause psychological exhaustion. Psychological exhaustion increases the likelihood that an individual will develop a variety of externalizing (such as aggressive and addictive behaviors) and internalizing (such as anxiety and depression) problems. The emergence of ego-depletion will reduce the psychological ownership of retired employees (Zell and Strickhouser, 2020) and affect their choice of reemployment intention. Based on this, this study introduces the concepts of ego-depletion and psychological ownership. This paper attempts to reveal the mediating effect of psychological ownership and explore how ego-depletion affects reemployment intention.

At the same time, social trust is also an important factor that triggers the intention of reemployment (Miller et al., 2021). Trust is a state of mind that is willing to take risks based on positive expectations of the friendly and kind intentions or actions of others. As a simplified mechanism of social complexity, social trust can establish a stable behavior expectation based on the existing information, so as to make up for the lack of information with a sense of security (Zhou and Cao, 2020). Social trust can improve individual confidence by reducing the cost of information acquisition, providing psychological channels, coordinating interpersonal cooperation, and promoting the effective organization and integration of social forces. Previous studies have shown that higher social trust is more likely to reduce the negative effect of ego-depletion and promote the reemployment choice of younger public sector retirees at the county level.

Existing literature has significantly enriched research on the reemployment of young retired public officials at the county level, providing essential theoretical support for this study. However, current research still presents areas warranting further exploration. Extensive studies have focused on individual characteristics influencing reemployment, such as age, gender, and health status; family factors, including family support and intergenerational relationships; and macro-level social institutional factors, such as pensions and employment policies. In contrast, systematic investigation into the deeper internal psychological mechanisms remains relatively insufficient. A notable gap exists in research that conceptualizes the reemployment of retired public officials as an ongoing adaptive process involving continuous interaction between internal psychological states and the external environment, subsequently integrating internal psychological depletion states—cognitive, emotional, and behavioral—with the external social trust environment for comprehensive examination. Consequently, the novelty of this study is primarily manifested in the following aspects. First, diverging from prior research emphasizing objective factors, this study introduces ego depletion theory into this field, operationalizing it into three dimensions—cognitive depletion, emotional depletion, and behavioral depletion—to systematically investigate its direct impact mechanism on reemployment behavior. Second, the concept of psychological ownership is innovatively introduced to construct and test its mediating pathway between ego depletion and reemployment behavior, thereby revealing the underlying psychological motivational mechanisms. Finally, social trust is incorporated as a moderating variable for the first time within the analytical framework to examine its moderating role in both the relationship between ego-depletion and psychological ownership, and between ego-depletion and reemployment behavior. This approach deepens the understanding of reemployment behavior among young retired county-level public officials within an integrated framework of individual internal psychological processes interacting with the external social environment. Based on empirical analysis in Jinan City, Shandong Province, this study aims to provide new insights and theoretical foundations for alleviating labor shortages caused by societal aging through the construction and validation of the proposed theoretical framework.

## 2 Theoretical analysis and research hypothesis

**Figure 1:**
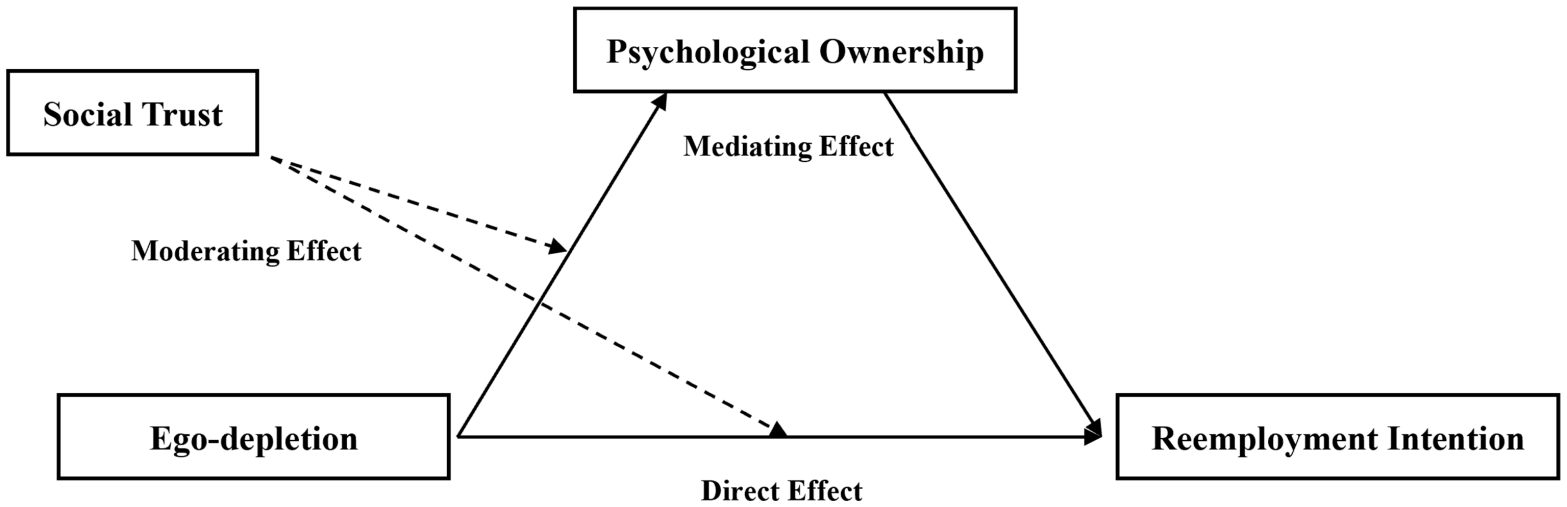
Theoretical framework.

To sum up, this paper constructed a research framework of “psychological ownership-ego-depletion-reemployment behavior” regulated by social trust (Figure 1). To study the effects of ego-depletion and psychological ownership on the reemployment behavior of the younger public sector retirees in counties. To explore the mediating mechanism of ego-depletion affecting the reemployment behavior of the younger public retirees through psychological ownership and the moderating mechanism of social trust among the three.

### 2.1 Direct effect of ego-depletion on reemployment intention

Ego-depletion refers to the state in which the individual’s self-control ability is exhausted after taking some actions that require the investment of self-control resources (Ming et al., 2020). Self-control is an individual’s self-inhibition of harmful reaction tendency and self-stimulation of beneficial reaction tendency through cognitive, emotional and behavioral strategies (Hunter et al., 2020). According to the resource model, various control activities such as inhibiting impulses, regulating emotions, and making difficult decisions draw on and deplete a single, limited pool of self-control resources. The present study adopts the view that ego-depletion is categorized into three distinct dimensions: cognitive, emotional, and behavioral depletion (Xia et al., 2020). Cognitive resources are considered as a kind of resources for individuals to carry out cognitive activities, which can help individuals to complete cognitive tasks smoothly. Cognitive depletion refers to the subjective cognition of an individual’s relative inferiority through social comparison. After the consumption of cognitive resources, the individual will hold a negative attitude towards work, including doubt of personal ability, envy of other people’s achievements, and decrease of self-identity. Emotional depletion means that it is difficult to maintain a normal working state physically and mentally due to emotional consumption. Emotional depletion is accompanied by psychological feelings such as tension, exhaustion, frustration and jealousy. Behavior depletion is the impulsive behavior manifested by the weakening of self-control ability after the consumption of self-control resources (Li et al., 2022), such as substance abuse (Mackey et al., 2020), over-consumption (Martijn et al., 2007), overeating, aggressive behavior (Yang and Tang, 2021), etc.

Ego-depletion is considered to significantly impair an individual’s psychological and behavioral functioning. Individuals experiencing ego depletion typically exhibit a range of negative consequences: increased negative affect, diminished information processing capacity, reduced prosocial behavior, lowered self-efficacy, pessimistic future expectations, and weakened behavioral intentions (Saleem et al., 2022). According to the loss spiral of Conservation of Resources Theory, ego-depletion undermines the psychological energy required to cope with challenges and pursue new goals, such as reemployment. Specifically, cognitive depletion leads to biased assessments of one’s own reemployment capabilities and opportunities, whereby individuals tend to underestimate their value. Emotional depletion reduces emotional resilience for coping with job-search stress and adapting to new environments. Behavioral depletion weakens the self-control necessary to overcome difficulties and sustain job-seeking activities. It can therefore be inferred that ego depletion significantly inhibits the reemployment behavior of young retirees from public service. Therefore, hypotheses are put forward:

*H1*: Ego-depletion has a negative effect on reemployment intention;

*H1a*: Cognitive depletion has a negative effect on reemployment intention;

*H1b*: Emotional depletion has a negative effect on reemployment intention;

*H1c*: Behavioral depletion has a negative effect on reemployment intention.

### 2.2 Mediating effect of psychological ownership

#### 2.2.1 Direct effect of ego-depletion on psychological ownership

Psychological ownership refers to an individual’s perception that a target object (material or non-material) or part of it is experienced as their own. Its core elements are possessiveness and the psychological connection to the target. This psychological state is primarily formed through control over the target, intimate knowledge of it, and self-investment into it. Control over objects is considered capable of generating a sense of ownership. Intimate knowledge arises when the target and the self become integrated through understanding, fostering ownership feelings. Self-investment occurs when greater dedication of time, attention, energy, and effort leads to stronger integration between the self and the target, thereby developing enhanced psychological ownership (Gieseler et al., 2021).

The retirement event itself constitutes a significant resource loss for public officials, stripping them of control over their former position and their proprietary identity. Resource Conservation Theory predicts that individuals experiencing resource loss threats or actual loss will strive to protect remaining resources and adopt a cautious stance toward future resource acquisition. Under ego depletion, the psychological resources available for establishing “intimate knowledge” of and investment into new objects (such as potential reemployment positions) become more limited (Ikeuchi et al., 2021). Regarding cognitive depletion, negative self-perceptions and self-doubt hinder the development of identification with and belongingness to new work roles, making it difficult to generate possessiveness (“this is my job”). In emotional depletion, persistent negative emotional states like tension and exhaustion reduce the willingness and energy required to explore new work environments and establish psychological connections, thereby inhibiting psychological ownership formation (Dang et al., 2017). While behavioral depletion manifests as weakened impulse control, its impact on psychological ownership may be more complex. Due to their professional characteristics, public officials’ deep-seated occupational identity and sense of responsibility may buffer the effect of behavioral depletion on establishing psychological ownership of new work, even when self-control resources are temporarily insufficient. Consequently, the direct negative impact of behavioral depletion on psychological ownership is likely weaker. Therefore, this study proposes the following hypotheses:

*H2*: Ego-depletion has a negative effect on psychological ownership

*H2a*: Cognitive depletion has a negative effect on psychological ownership

*H2b*: Emotional depletion has a negative effect on psychological ownership

*H2c*: Behavioral depletion has a negative effect on psychological ownership

#### 2.2.2 Direct effect of psychological ownership on reemployment intention

Psychological ownership is when an individual feels the object (material or immaterial) or part of the object as if it were his own (Schilke et al., 2021). The core of psychological ownership is the sense of possession and the psychological connection with the object (Wang et al., 2022). Pierce et al. (2003) pointed out that psychological ownership can be formed in three ways: control the goal, close understanding, and self-involvement. Control goal means that the control of the goal can produce the sense of ownership of the goal. Close understanding means that when an individual understands the goal, the goal and the self will merge, resulting in a sense of ownership of the goal. The more time, attention, energy and effort an individual puts into a goal, the more closely his or her self integrates with the goal and develops a stronger psychological ownership. Firstly, psychological ownership of work creates a sense of being the “owner” among retired public officials. This not only enhances their confidence in task competence but also reinforces the positive significance of work for self-realization, strengthening self-efficacy and perceived value. Secondly, psychological ownership fosters a sense of responsibility and affective commitment towards work among retired public officials. This promotes a more positive view of participation’s value and generates intrinsic motivation to maintain and contribute to the work. Finally, strong feelings of psychological connection and responsibility are key intrinsic motivators driving retired public officials to take action. Those with high psychological ownership are more likely to proactively seek reemployment opportunities to achieve sustained contribution and value continuation.

Public sector personnel have been engaged in relevant work for a long time, and have paid a lot of time, energy and effort for their work, so they can skillfully use corresponding knowledge to solve work problems. The single, repeatable work they have been engaged in for a long time makes them have a sense of belonging to work and a strong psychological ownership. Management ownership can make public sector retirees more willing to remain members of an organization and improve individual self-efficacy (Pierce et al., 2003). Retirement, on the other hand, means taking away the sense of ownership. The gap of not being able to continue working will be difficult for some public sector personnel to accept, resulting in a sense of depletion of psychological ownership. Previous studies have found that psychological ownership has a positive correlation with employee retention intention and a negative correlation with turnover intention (Sepeng et al., 2020; Jing and Yan, 2022). Studies have shown that psychological ownership has an impact on employees’ work attitude, such as psychological ownership and employees’ organizational commitment (Peck et al., 2021), job satisfaction, and retention intention (Kavvouris et al., 2020). Psychological ownership can explain the internal motivation of the reemployment intention of the younger public sector retirees. When public sector employees have psychological ownership of work and can obtain satisfaction and sense of gain from work, it will have a positive impact on self-efficacy, attitude and willingness to reemployment. Based on this, the following hypothesis is proposed:

*H3*: Psychological ownership has a positive effect on reemployment intention

#### 2.2.3 Mediating effect of psychological ownership

Studies have shown that individuals with high personal subjective cognition, positive emotions and high autonomy will have less ego-depletion (Li et al., 2022). Specifically, individuals with high subjective cognition can mitigate the after-effects of ego-depletion by perceiving current behavior as beneficial for the future (Oeri and Roebers, 2020). If individuals know that their efforts are helpful to others or themselves, they can enhance their commitment to the organization, resulting in altruistic behavior, organizational citizenship behavior, butler behavior and other positive behavioral consequences (Hagger et al., 2019). Individuals with positive emotions can compensate for ego-depletion, promote self-control (She et al., 2022), avoid being influenced by external circumstances, and strengthen their inner choices. Individuals with high autonomy tendency have a strong need to complete personal goals independently, and will make every effort to reduce the possibility of failure, improve individual toughness and enhance the ability to withstand pressure. However, individuals with high psychological ownership will produce a spirit of dedication, and will produce more organizational citizenship behaviors, which will provide rich working resources for individuals (Rodriguez Montalban et al., 2014). Therefore, when public sector retirees experience cognitive, emotional, or behavioral depletion that shake their willingness to re-enter the workforce, if individuals have both rich working resources (autonomy) and rich personal resources (psychological ownership), they can greatly reduce the level of depletion and enhance their personal beliefs. Therefore, psychological ownership can alleviate the negative effects of ego-depletion and generate positive reemployment intentions.

Psychological ownership serves as the key psychological mechanism through which the negative effects of ego-depletion are transmitted. Individuals with higher psychological ownership exhibit greater psychological resilience due to stronger autonomy tendencies, positive affect, and perceived goal value. This enhanced resilience enables more effective buffering against the detrimental impact of ego-depletion, thereby maintaining active reemployment intentions and behaviors. Consequently, the following hypothesis is proposed:

*H4*: Psychological ownership has a positive mediating effect between ego-depletion and reemployment intention;

*H4a*: Psychological ownership has a positive mediating effect between cognitive depletion and reemployment intention;

*H4b*: Psychological ownership has a positive mediating effect on the effect of emotional depletion on reemployment intention;

*H4c*: Psychological ownership has a positive mediating effect between behavioral depletion and reemployment intention.

### 2.3 Moderating effect of social trust

#### 2.3.1 The moderating effect of social trust on ego-depletion and psychological ownership

Social support theory posits that environments characterized by high social trust indicate the perception of a more reliable social support network by individuals (Yang, 2019). Such supportive resources can partially offset psychological resources depleted through ego-depletion, providing an essential psychological “safety net” and energy replenishment (Xiao et al., 2021). This facilitates engagement with new environments through understanding, control, and self-investment, thereby contributing to the establishment of psychological ownership.

Social exchange theory suggests that heightened trust reduces perceived complexity and potential risks within re-employment settings (Seo and Park, 2020). When institutions are perceived as fair and others as trustworthy, the perceived risk and effort required to establish psychological connections with new work roles during states of ego-depletion are diminished (Urena et al., 2019). This mitigates the inhibitory effect of depleted states on psychological engagement.

Furthermore, trust in institutions including re-employment policy safeguards and environmental factors such as equitable opportunity provides anticipated external stability and control (Balliet and Van Lange, 2013). This partially compensates for the loss of control stemming from internal depletion, sustaining motivation to develop psychological ownership even when personal resources are temporarily insufficient. Consequently, higher levels of social trust are expected to weaken the negative impact of ego-depletion on psychological ownership (Siegrist, 2021). Hence, it is hypothesized that:

*H5*: Social trust has a positive moderating effect between ego-depletion and psychological ownership

*H5a*: Social trust has a positive moderating effect between cognitive depletion and psychological ownership

*H5b*: Social trust has a positive moderating effect between emotional depletion and psychological ownership

*H5c*: Social trust has a positive moderating effect between behavioral depletion and psychological ownership

#### 2.3.2 The moderating effect of social trust on ego-depletion and reemployment intention

Social trust is shaped by long-term cultural accumulation and results from rational assessments and choices regarding personal gains and losses (Liu and Gong, 2020). It serves as the foundational value for social cohesion and solidarity. The composition and level of social trust influence the degree of societal engagement. Consequently, social trust can be reflected in the level of social quality (Jiang et al., 2020). Cognitive Evaluation Theory posits that individuals in high-trust environments are more likely to attribute ego-depletion states—such as self-doubt or low mood—to temporary, external factors rather than permanent personal deficiencies (Irawan et al., 2022). High institutional trust, including confidence in safeguards like age-discrimination laws, and high moral trust, reflected in societal norms such as respect for elders, are suggested to mitigate the perceived threat of reemployment barriers (Zhou and Guo, 2020). This mitigation buffers the direct negative impact of ego-depletion on reemployment confidence and intention (Huff and Kelley, 2003).

Furthermore, strong institutional trust indicates a belief in effective policy support and legal protections, while strong moral trust signifies an expectation of fair opportunities and an inclusive social environment (Zhang et al., 2018; Rajkumar et al., 2022). These perceptions of trust are theorized to provide alternative resources and positive behavioral cues for coping with ego-depletion (Ichihashi et al., 2020). Consequently, they may directly motivate or sustain the behavioral drive to overcome internal depletion and pursue reemployment. Consequently, the following hypothesis is proposed:

*H6*: Social trust has a positive moderating effect on ego-depletion and reemployment intention;

*H6a*: Social trust has a positive moderating effect on cognitive depletion and reemployment intention

*H6b*: Social trust has a positive moderating effect on emotional depletion and reemployment intention;

*H6c*: Social trust has a positive moderating effect on behavioral depletion and reemployment intention.

## 3 Methods

### 3.1 Respondents

In this study, public sector employees who have retired for less than 10 years in Jinan City of Shandong Province are investigated. The public sector personnel in this study refers to the staff who perform public duties and engage in public affairs according to law. According to the specific situation of Jinan city, the staff of government agencies, enterprises and public institutions and community neighborhood committees are selected for research. According to the Bulletin of the 7th National Population Census of Jinan City, the resident population of Jinan city is 9.202,400, accounting for 9.06% of the province’s population. The population is huge. Compared with the data of the sixth Census, the share of the population aged 60 and above increased by 5.83 percentage points, and that of the population aged 65 and above increased by 4.76 percentage points. The number of elderly population showed an upward trend. As the capital city of Shandong Province, Jinan has always been a pilot city for policy exploration in Shandong Province. Therefore, the research conclusion based on Jinan as the investigation site has reference value.

The reasons for selecting public sector workers who have been retired for less than 10 years are as follows: On the one hand, according to the Bulletin of the Seventh National Population Census, among the elderly aged 60 and above in China, the younger elderly aged 60–69 account for 55.83%. Most of them have the advantage of knowledge, experience, skills and good physical condition. On the other hand, from the perspective of retirement age structure, China’s current retirement age of workers is 60 years old for men, 55 years old for women cadres, and 50 years old for women workers. The “Healthy China 2030” plan proposes that the average life expectancy will reach 79.0 years by 2030. With the increase in the level of education of the whole population, the age at which workers begin to work has been significantly delayed. It can be said that young elderly talents are important human resources that cannot be ignored at present, and effectively promoting the reemployment of elderly talents is the future development trend of society.

Among 1,036 public sector workers, 560 were male (54.1%) and 476 were female (45.9%). In terms of academic qualifications, 280 college students or below (27%), 467 bachelor students (45.1%), 260 master students (25.1%), 29 doctor students (2.8%); More than 60% have been retired for less than 5 years (74.8%); In terms of marital status, 11 were unmarried (1.1%), 974 were married (94%), 32 were divorced (3.1%), and 19 were widowed (1.8%) (Tables 1, 2).

**Table 1:** Descriptive statistic results.

Items	Categories	Frequency	Percentage
Sex	Male	560	54.1
	Female	476	45.9
Education background	Junior College and Below	280	27.0
	Undergraduate	467	45.1
	Postgraduate	260	25.1
	Doctor	29	2.8
Retirement time	Less than 1 year	130	12.6
	1–3 years	305	29.4
	3–5 years	340	32.8
	5–10 years	261	25.2
Marital status	Unmarried	11	1.1
	married	974	94.0
	divorced	32	3.1
	widowed	19	1.8
Physical condition	Very unhealthy	11	1.1
	Not healthy	32	3.1
	general	104	10.0
	Relatively healthy	363	35.0
	Very healthy	526	50.8
Reemployment will	yes	912	88.0
	no	124	12.0
Reemployment attitude	Very low confidence	34	3.3
	Less confident	93	9.0
	normal	177	17.1
	More confident	461	44.5
	Very confident	271	26.2
Expected monthly salary	1,500–2000 yuan	260	25.1
	2000–3,500 yuan	436	42.1
	3,500–5,000 yuan	187	18.1
	More than 5,000 yuan	153	14.8

**Table 2:** Satisfaction evaluation results.

Items Frequency categories	Very dissatisfied	Not satisfied	General	Quite satisfactory	Very satisfied
Working state	29 (2.8)	86 (8.3)	144 (13.9)	535 (51.6)	242 (23.4)
Life satisfaction	43 (4.2)	78 (7.5)	170 (16.4)	392 (37.8)	353 (34.1)
Job competence	33 (3.2)	83 (8.0)	142 (13.7)	535 (51.6)	243 (23.5)
Job ability	35 (3.4)	88 (8.5)	174 (16.8)	503 (48.6)	236 (22.8)
Job confidence	26 (2.5)	86 (8.3)	188 (18.1)	498 (48.1)	238 (23.0)

### 3.2 Research tools

Based on domestic and foreign maturity scales, combined with research objectives, psychological ownership, ego-depletion, social trust, reemployment intention scales are designed. A 5-point Likert scale was used for each variable, with 1–5 representing “strongly disagree,” “disagree,” “general,” “agree” and “strongly agree” respectively.

#### 3.2.1 Independent variable: ego-depletion

Based on the relevant research conducted by Ying et al. (2023) and Tang et al. (2022), the questionnaire was designed to divide ego depletion into three dimensions: cognitive depletion, behavioral depletion and emotional depletion. There are 11 items.

#### 3.2.2 Mediating variable: psychological ownership

Psychological ownership was divided into three core variables: psychological ownership, psychological control and psychological identity. The scale mainly draws on the relevant measurement scales in the existing research, and makes appropriate modifications according to the research situation. Sense of ownership refers to the measurement scale of Atatsi et al. (2021), which has good reliability and validity and is the most representative scale for measuring psychological ownership. The items of the sense of Control scale are derived from the sociopolitical control scale in Alexander L’s (Lithopoulos et al., 2023) research on perceptual control in a specific field. The scale of identity is based on the empirical study of Zhang et al. (2023), which has high reliability and validity.

#### 3.2.3 Dependent variable: reemployment intention

With reference to the relevant studies of Thomassen et al. (2022), this study is deleted and modified according to the actual situation of this study. Indicators were selected from three dimensions: self-efficacy, attitude and intention. There are 14 items in the scale.

#### 3.2.4 Moderating variable: social trust

According to the relevant studies of Cai et al. (2023) and Kondo et al. (2023), the social trust was measured from two dimensions of institutional trust and interpersonal trust. System trust includes two sub-dimensions: policy trust and legal trust. Interpersonal trust includes two sub-dimensions: urban environment and social atmosphere. There are 16 items in the scale.

### 3.3 Programs

The data were derived from a sample survey of younger public sector retirees in Jinan, Shandong Province from October to December 2023. The research adopts the method of stratified step-by-step sampling and random sampling to select samples. First of all, five districts were randomly selected in Jinan with the district as the primary sampling unit. Subsequently, 3 to 4 public sector employees were selected in each district and a list of eligible public sector retirees was obtained after communication with the department head. Finally, after getting in touch with the corresponding younger public sector retirees and obtaining their consent, questionnaires were distributed through a combination of online and offline. 1,200 questionnaires were sent out, and 1,036 valid samples were obtained, with an effective rate of 86.3%.

### 3.4 Data analysis

In this study, SPSS 22.0 and AMOS 26.0 were used to make an empirical analysis of the data. Structural equation model was used to conduct an empirical study on the influence of ego-depletion of younger public sector retirees on their reemployment intention and its mechanism. Meanwhile, the regression path analysis method of Bootstrap was used to bring four variables into the same model to test the significance of the mediating effect and the moderating effect. All study variables were normalized before model testing. The testing of the moderating effect mainly employed hierarchical multiple regression, with two multiple regression models primarily established. The first model incorporates the independent variable and the moderating variable. This model is designed to examine whether the independent variable and the moderating variable exert an influence on the dependent variable, and to determine the explanatory power of the model—specifically, to assess the magnitude of the model’s *R*^2^. The second model incorporates the independent variable, the moderating variable, and the interaction term between the independent variable and the moderating variable. If the regression coefficient of the interaction term is significant and the model’s R^2^ increases significantly, it indicates that the moderating variable exerts a significant moderating effect on the relationship between the independent variable and the dependent variable.

## 4 Results

### 4.1 Descriptive statistics and correlation analysis

Among the 6 latent variables involved in this paper (except that upward social comparison tendency had no significant correlation with behavioral depletion, *p* > 0.05), the pairwise positive correlation of other latent variables was significant (*p* < 0.01) (Table 3).

**Table 3:** Descriptive statistics and correlation analysis (*N* = 1,036).

Variable	*M*	SD	1	2	3	4	5	6
1. Cognitive Depletion	2.440	0.948	1					
2. Emotional Depletion	2.444	0.971	0.337**	1				
3. Behavioral Depletion	2.480	0.928	0.326**	0.281**	1			
4. Psychological Ownership	3.573	0.654	−0.332**	−0.292**	−0.191**	1		
5. Reemployment Intention	3.589	0.640	−0.385**	−0.400**	−0.349**	0.405**	1	
6. Social Trust	3.828	0.647	−0.091**	−0.167**	−0.074*	0.181**	0.149**	1

*P < 0.05, **P < 0.01, ***P < 0.001.

### 4.2 Structural equation model test results

#### 4.2.1 Reliability and validity test

Cronbach’s Alpha of the overall scale of ego-depletion was 0.849. Cronbach’s Alpha of cognitive depletion, emotional depletion, and behavioral depletion were 0.834, 0.864, and 0.864, respectively, all of which met the basic standard of greater than 0.7. Cronbach’s Alpha of psychological ownership, reemployment intention and social trust were 0.905, 0.873, and 0.909, respectively, which were greater than 0.7. It can be seen that the questionnaire scale used in this study has good reliability. In addition, the CITC between the observed variable and its latent variable was greater than 0.5, indicating good reliability of the questionnaire.

The KMO test value of the survey data was 0.911 (greater than 0.70), indicating that the questionnaire was suitable for factor analysis. Bartlett sphericity test results showed that the approximate chi-square value was 11191.48, the significance probability was 0.000 (*p* < 0.01), and the validity structure was good. In the process of factor analysis, 11 common factors with eigenvalues greater than 1 were extracted by principal component analysis, and the total variance explanation rate was all greater than 60%, indicating good validity of the scale. Factor rotation was performed by orthogonal rotation with maximum variance method, and 59 question options were classified into 11 factors. The load of each measurement item was higher than 0.5, and the questionnaire content validity met the requirements. The results of confirmatory factor analysis show that the standardized factor load of each item was greater than 0.5, and the standard error value of S.E was less than 0.5. The results of exploratory factor analysis indicated that the validity of the questionnaire met the requirements. Finally, AVE of each dimension was greater than 0.5, and its square root was greater than the correlation coefficient between variables, indicating that the convergent validity of the scale met the requirements (Carlson and Herdman, 2012).

#### 4.2.2 Structural equation model fitting index

**Figure 2:**
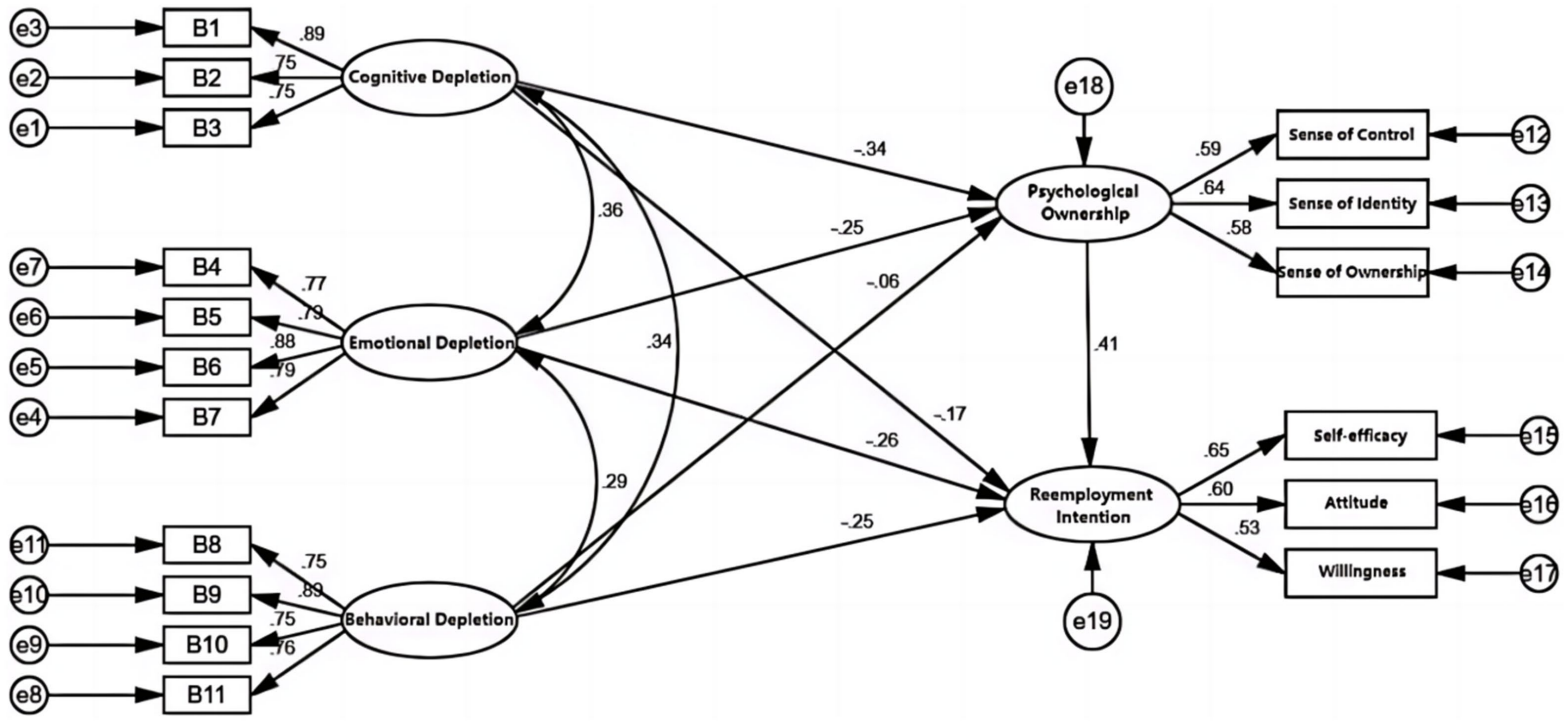
Structural equation model diagram.

The results show that the X2/df value was 3.291, less than 5; The RMSEA was 0.047, which was less than the standard level 0.08, indicating a good fit. GFI = 0.962, AGFI = 0.946, NFI = 0.951, IFI = 0.965, CFI = 0.965, TLI = 0.957, all the indicators of goodness of fit reached the general standard. It showed that the structural equation model established in this study was effective and well matched with the recovered data (Figure 2).

### 4.3 Path analysis result

The standardized path coefficient of cognitive depletion orientation on reemployment intention was −0.168 (*t* = −3.728, *p* < 0.001), indicating that cognitive depletion orientation had a significant negative effect on reemployment intention. Therefore, H1a was assumed to be true; The standardized path coefficient of emotional depletion on reemployment intention was −0.259 (*t* = −6.147, *p* < 0.001), indicating that emotional depletion had a significant negative effect on reemployment intention. Therefore, H1b was assumed to be established; The standardized path coefficient of behavioral depletion on reemployment intention was −0.248 (*t* = −6.228, *p* < 0.001), indicating that behavioral depletion had a significant negative effect on reemployment intention. Therefore, H1c was assumed to be true.

The standardized path coefficient of cognitive depletion orientation on psychological ownership was −0.344 (*t* = −7.118, *p* < 0.001), indicating that cognitive depletion orientation had a significant negative effect on psychological ownership. Therefore, H2a was assumed to be true; The standardized path coefficient of emotional depletion on psychological ownership was −0.248 (*t* = −5.564, *p* < 0.001), indicating that emotional depletion had a significant negative effect on psychological ownership. Therefore, H2b was assumed to be true; The standardized path coefficient of behavioral depletion on psychological ownership was −0.058 (*t* = −1.374, *p* > 0.05), indicating that behavioral depletion had no significant effect on psychological ownership. So the hypothesis H2c was not true.

The standardized path coefficient of psychological ownership on reemployment intention was 0.411 (*t* = 7.018, *p* < 0.001), indicating that psychological ownership had a significant positive effect on reemployment intention. Therefore, it was assumed that H3 was valid (Table 4).

**Table 4:** Pathway analysis result.

Pathway			Standardized path coefficient	S.E.	C.R.	*P*
Psychological ownership	<−--	Cognitive depletion	−0.344	0.03	−7.118	***
Psychological ownership	<−--	Emotional depletion	−0.248	0.024	−5.564	***
Psychological ownership	<−--	Behavioral depletion	−0.058	0.025	−1.374	0.169
Reemployment intention	<−--	Cognitive depletion	−0.168	0.032	−3.728	***
Reemployment intention	<−--	Emotional depletion	−0.259	0.025	−6.147	***
Reemployment intention	<−--	Behavioral depletion	−0.248	0.027	−6.228	***
Reemployment intention	<−--	Psychological ownership	0.411	0.065	7.018	***

*P < 0.05, **P < 0.01, ***P < 0.001.

### 4.4 Mediating effect

The results of the mediation effect test showed that the total effect value of the mediation path [cognitive depletion – reemployment intention] was −0.309. The upper and lower sections of the 95% confidence interval were negative, excluding 0, and the P was less than the significant level 0.05. It showed that the total effect exists. The direct effect size was −0.168. Both the upper and lower sections of the 95% confidence interval were negative, excluding 0, and the P was less than the significant level 0.05. The direct effect existed, accounting for 54.4% of the total effect. The indirect effect (cognitive depletion - psychological ownership - reemployment intention) was −0.141. The upper and lower sections of the 95% confidence interval were negative, excluding 0, and the P was less than the significant level 0.05. The indirect effect existed, accounting for 45.6% of the total effect. Therefore, it was proved that hypothesis H4a was valid.

In the mediation path [emotional depletion - reemployment intention], the total effect size was −0.361. The upper and lower sections of the 95% confidence interval were negative, excluding 0, and the P was less than the significant level 0.05. It showed that the total effect exists. The direct effect value was −0.259. Both the upper and lower sections of the 95% confidence interval were negative, excluding 0, and the P was less than the significant level 0.05. The direct effect existed, accounting for 71.7% of the total effect. The indirect effect (cognitive depletion – psychological ownership – reemployment intention) was −0.102. The upper and lower sections of the 95% confidence interval were negative, excluding 0, and the P was less than the significant level 0.05. The indirect effect existed, accounting for 28.3% of the total effect. Therefore, it was proved that hypothesis H4b was valid.

In the mediation path [behavioral depletion-reemployment intention], the total effect size was −0.272. The upper and lower sections of the 95% confidence interval were negative, excluding 0, and the P was less than the significant level 0.05. It showed that the total effect exists. The direct effect size was −0.248. Both the upper and lower sections of the 95% confidence interval were negative, excluding 0, and the P was less than the significant level 0.05. The direct effect existed, accounting for 91.2% of the total effect. The indirect effect [cognitive depletion – psychological ownership – reemployment intention] was −0.024. The upper and lower sections of 95% confidence interval were both negative and positive, including 0, and the P was greater than the significant level 0.05. This indicated that the indirect effect does not exist, so hypothesis H4c was proved to be invalid (Table 5).

**Table 5:** Bootstrap for mediating effect test.

Effect pathway	Effect type	Effect value	SE	95% Confidence interval		*P*	Effect ratio
				Lower interval	Upper interval		
Cognitive depletion—Reemployment intention	Total Effect	−0.309	−0.394	−0.225	0.000	-	−0.309
Cognitive depletion—Reemployment intention	Direct Effect	−0.168	−0.262	−0.074	0.001	54.4%	−0.168
Cognitive depletion—Psychological ownership—Reemployment intention	Indirect Effect	−0.141	−0.204	−0.091	0.000	45.6%	−0.141
Emotional depletion—Reemployment intention	Total Effect	−0.361	−0.444	−0.278	0.000	-	−0.361
Emotional depletion—Reemployment intention	Direct Effect	−0.259	−0.353	−0.169	0.000	71.7%	−0.259
Emotional depletion—Psychological ownership—Reemployment intention	Indirect Effect	−0.102	−0.158	−0.059	0.000	28.3%	−0.102
Behavioral depletion—Reemployment intention	Total Effect	−0.272	−0.359	−0.192	0.000	-	−0.272
Behavioral depletion—Reemployment intention	Direct Effect	−0.248	−0.336	−0.162	0.000	91.2%	−0.248
Behavioral depletion—Psychological ownership—Reemployment intention	Indirect Effect	−0.024	−0.071	0.014	0.199	8.8%	−0.024

### 4.5 Moderating effect

#### 4.5.1 The moderating effect of social trust on cognitive depletion and psychological ownership

In Table 6, Model 1 established a multiple regression model with cognitive depletion and social trust as independent variables and psychological ownership as dependent variables. Model 2 established a multiple regression model with cognitive depletion, social trust and interaction item cognitive depletion * social trust as independent variables and psychological ownership as dependent variables. In model 1, cognitive depletion of independent variable had a significant negative effect on psychological ownership (*β* = −0.318, *t* = −10.921, *p* < 0.001). In Model 2, the regression coefficient of the interaction term between the independent variable and the regulating variable was 0.134 (*t* = 3.443, *p* < 0.01), indicating that the interaction term had a significant positive effect on psychological ownership. In addition, the *R*^2^ of model 1 was 0.133, and that of model 2 was 0.143, which was significantly improved, indicating that the interpretation ability of the model was enhanced. Therefore, it was proved that the moderating variable social trust had a significant positive moderating effect on the relationship between cognitive depletion and psychological ownership. Therefore, it was assumed that H5a was valid (Table 6).

**Table 6:** The moderating effect of social trust on cognitive depletion and psychological ownership.

Variable	Model 1		Model 2	
	*β*	*t*	*β*	*t*
Cognitive depletion	−0.318	−10.921***	−0.312	−10.761***
Social Trust	0.152	5.212***	0.242	6.194***
Cognitive depletion*Social trust	–	–	0.134	3.443**
*R* ^2^	0.133		0.143	
Adjusted *R*^2^	0.131		0.140	
Δ*R*^2^	0.133		0.010	
*F*	79.049***		57.205***	

*P < 0.05, **P < 0.01, ***P < 0.001.

#### 4.5.2 The moderating effect of social trust on emotional depletion and psychological ownership

In Table 7, Model 1 established a multiple regression model with emotional depletion and social trust as independent variables and psychological ownership as dependent variables. In Model 2, a multiple regression model was established with emotional depletion, social trust and interaction item emotional depletion * social trust as independent variables and psychological ownership as dependent variables. In model 1, independent variable emotional wear had a significant negative effect on psychological ownership (*β* = −0.27, *t* = −9.030, *p* < 0.001). In model 2, the regression coefficient of the interaction term between the independent variable and the regulating variable was 0.106 (*t* = 2.811, *p* < 0.01), indicating that the interaction term had a significant positive effect on psychological ownership. In addition, the *R*^2^ of model 1 was 0.103, and that of model 2 was 0.110, which was significantly improved, indicating that the interpretation ability of the model was enhanced. Therefore, it was proved that the moderating variable social trust had a significant positive moderating effect on the relationship between emotional depletion and psychological ownership. Therefore, it was assumed that H5b was true (Table 7).

**Table 7:** The moderating effect of social trust on emotional depletion and psychological ownership.

Variable	Model 1		Model 2	
	*β*	*t*	*β*	*t*
Emotional depletion	−0.27	−9.030***	−0.275	−9.218***
Social trust	0.136	4.538***	0.2	5.321***
Emotional depletion*Social trust	–	–	0.106	2.811**
*R* ^2^	0.103		0.110	
Adjusted *R*^2^	0.102		0.108	
Δ*R*^2^	0.103		0.010	
*F*	59.546***		42.597***	

*P < 0.05, **P < 0.01, ***P < 0.001.

#### 4.5.3 The moderating effect of social trust between behavior depletion and psychological ownership

In Table 8, Model 1 established a multiple regression model with behavioral depletion and social trust as independent variables and psychological ownership as dependent variables. In Model 2, a multiple regression model was established with behavior depletion, social trust and interaction item behavior depletion * social trust as independent variables and psychological ownership as dependent variables. In model 1, the behavior depletion of independent variable had a significant negative effect on psychological ownership (*β* = −0.179, *t* = −5.928, *p* < 0.001). In Model 2, the regression coefficient of the interaction term between the independent variable and the regulating variable was 0.099 (*t* = 2.378, *p* < 0.01), indicating that the interaction term had a significant positive effect on psychological ownership. In addition, the *R*^2^ of model 1 was 0.064, and that of model 2 was 0.070, which was significantly increased, indicating that the interpretation ability of the model was enhanced. Therefore, it was proved that the moderating variable social trust had a significant positive moderating effect on the relationship between behavior depletion and psychological ownership. Therefore, it was assumed that H5c was true (Table 8).

**Table 8:** The moderating effect of social trust between behavior depletion and psychological ownership.

Variable	Model 1		Model 2	
	*β*	*t*	*β*	*t*
Behavioral depletion	−0.179	−5.928***	−0.176	−5.827***
Social trust	0.167	5.547***	0.236	5.661***
Behavioral depletion*Social Trust	–	–	0.099	2.378*
*R* ^2^	0.064		0.070	
Adjusted *R*^2^	0.063		0.067	
Δ*R*^2^	0.064		0.010	
F	35.565***		25.702***	

*P < 0.05, **P < 0.01, ***P < 0.001.

#### 4.5.4 The moderating effect of social trust on cognitive depletion and reemployment intention

In Table 9, Model 1 established a multiple regression model with cognitive depletion and social trust as independent variables and reemployment intention as dependent variables. In Model 2, a multiple regression model was established with cognitive depletion, social trust and interaction item cognitive depletion * social trust as independent variables, and reemployment intention as dependent variables. In model 1, cognitive depletion of independent variable had a significant negative effect on reemployment intention (*β* = −0.374, *t* = −13.081, *p* < 0.001). In Model 2, the regression coefficient of the interaction term between the independent variable and the regulating variable was 0.144 (*t* = 3.757, *p* < 0.001), indicating that the interaction term had a significant positive effect on reemployment intention. In addition, the *R*^2^ of model 1 was 0.161, and that of model 2 was 0.172, which was significantly improved, indicating that the interpretation ability of the model was enhanced. Therefore, it was proved that the moderating variable social trust had a significant positive moderating effect on the relationship between cognitive depletion and reemployment intention. So suppose that H6a was true (Table 9).

**Table 9:** The moderating effect of social trust on cognitive depletion and reemployment intention.

Variable	Model 1		Model 2	
	β	t	β	t
Cognitive depletion	−0.374	−13.081***	−0.368	−12.925***
Social trust	0.115	4.015***	0.212	5.517***
Cognitive depletion*Social trust	-	-	0.144	3.757***
*R* ^2^	0.161		0.172	
Adjusted *R*^2^	0.160		0.170	
Δ*R*^2^	0.161		0.011	
*F*	99.216***		71.689***	

*P < 0.05, **P < 0.01, ***P < 0.001.

#### 4.5.5 The moderating effect of social trust on emotional depletion and reemployment intention

In Table 10, Model 1 established a multiple regression model with emotional wear and social trust as independent variables and reemployment intention as dependent variables. In Model 2, a multiple regression model was established with emotional depletion, social trust and interaction item emotional depletion * social trust as independent variables and reemployment intention as dependent variables. In model 1, independent variable emotional wear had a significant negative effect on reemployment intention (*β* = −0.386, *t* = −13.413, *p* < 0.001). In Model 2, the regression coefficient of the interaction term of the independent variable and the regulating variable was 0.066 (*t* = 1.813, *p* > 0.05), indicating that the interaction term had no significant effect on reemployment intention. And the *R*^2^ of model 1 was 0.167, and the *R*^2^ of model 2 was 0.170, which was significantly improved. Therefore, it was proved that the moderating variable social trust had no significant moderating effect on the relationship between emotional depletion and reemployment intention. So suppose that H6b was not true (Table 10).

**Table 10:** The moderating effect of social trust on emotional depletion and reemployment intention.

Variable	Model 1		Model 2	
	*β*	*t*	*β*	*t*
Emotional depletion	−0.386	−13.413***	−0.389	−13.514***
Social trust	0.085	2.938**	0.125	3.435**
Emotional depletion*Social trust	-	-	0.066	1.813
*R* ^2^	0.167		0.170	
Adjusted *R*^2^	0.166		0.167	
Δ*R*^2^	0.167		0.003	
*F*	103.704***		70.385***	

*P < 0.05, **P < 0.01, ***P < 0.001.

#### 4.5.6 The moderating effect of social trust on behavior depletion and reemployment intention

In Table 11, Model 1 establishes a multiple regression model with behavioral depletion and social trust as independent variables and reemployment intention as dependent variables. In Model 2, a multiple regression model was established with behavior depletion, social trust and interaction item behavior depletion * social trust as independent variables and reemployment intention as dependent variables. In model 1, the behavior depletion of independent variable had a significant negative effect on reemployment intention (*β* = −0.340, *t* = −11.723, *p* < 0.001). In Model 2, the regression coefficient of the interaction term between the independent variable and the regulating variable was 0.146 (*t* = 3.671, *p* < 0.001), indicating that the interaction term had a significant positive effect on reemployment intention. In addition, the R^2^ of model 1 was 0.137, and that of model 2 was 0.148, which was significantly improved, indicating that the interpretation ability of the model was enhanced. Therefore, it was proved that the moderating variable social trust had a significant positive moderating effect on the relationship between behavior depletion and reemployment intention. Therefore, it was assumed that H6c was true (Table 11).

**Table 11:** The moderating effect of social trust on behavior depletion and reemployment intention.

Variable	Model 1		Model 2	
	*β*	*t*	*β*	*t*
Behavioral depletion	−0.340	−11.723***	−0.335	−11.614***
Social trust	0.124	4.276***	0.225	5.646***
Behavioral depletion*Social trust	–	–	0.146	3.671***
*R* ^2^	0.137		0.148	
Adjusted *R*^2^	0.135		0.146	
Δ*R*^2^	0.137		0.011	
*F*	81.982***		59.807***	

*P < 0.05, **P < 0.01, ***P < 0.001.

## 5 Discussion and policy recommendation

### 5.1 Discussion

This paper focuses on the reemployment of younger public sector retirees in counties under the background of aging, and constructed a theoretical framework of “psychological ownership – ego-depletion – reemployment intention” moderated by social trust. Based on the micro-survey data of 1,036 younger public sector retirees in Jinan, Shandong Province, exploratory factor analysis and Bootstrap moderated mediation test method were applied. This paper empirically explored the internal mechanism of the effects of various dimensions of ego-depletion (cognitive depletion, emotional depletion, behavioral depletion) on the reemployment intention of the younger public sector retirees in counties. The study found:

First, ego-depletion has a negative effect on reemployment intention. All dimensions of ego-depletion significantly inhibited reemployment behavior among low-aged retired public officers, with effect strengths ranked as follows: cognitive depletion > behavioral depletion > emotional depletion. This indicates that ego-depletion reduces psychological capital, elevates anxiety levels, and exacerbates cognitive biases, leading to underestimation of personal capabilities and environmental control, along with pessimistic future expectations. Consequently, enthusiasm, confidence, and behavioral intentions toward reemployment are significantly undermined, potentially triggering negative emotions such as anxiety and depression.

Second, differential effects of ego-depletion on psychological ownership were identified. Both cognitive depletion and emotional depletion exerted significant negative impacts on psychological ownership. This suggests that depletion at cognitive and emotional levels diminishes willingness for intimate understanding of work and reduces time and energy investment, thereby weakening perceived control and identification with work roles—manifested as reduced psychological ownership (Schnabel and Pollatos, 2022). However, no significant direct effect of behavioral depletion on psychological ownership was substantiated. This outcome may relate to the unique characteristics of public officers, where inherent public service motivation and professional norms, such as strong civic ethos and democratic spirit, may sustain deep-seated role identification and responsibility despite temporary behavioral control deficits (Zhang et al., 2019).

Third, the mediating role of psychological ownership in specific ego-depletion pathways was verified. Psychological ownership partially mediated the relationship through which cognitive depletion and emotional depletion inhibit reemployment behavior. Specifically, cognitive and emotional depletion not only directly suppress reemployment behavior but also indirectly inhibit it by undermining psychological ownership—a key psychological mechanism. This reflects that high psychological ownership enhances subjective cognitive clarity, positive emotional states, and autonomous tendencies, thereby strengthening psychological resilience and partially buffering the negative impacts of cognitive and emotional depletion. Nevertheless, no mediating effect of psychological ownership was observed between self-behavioral depletion and reemployment behavior. This may stem from age-related physiological and psychological decline among low-aged retired public officers, wherein psychological ownership (“willingness”) fails to mitigate behavioral depletion’s direct impediment to reemployment due to practical capacity limitations (“ability constraints”).

Fourth, complex moderating effects of social trust were revealed. Social trust positively moderated the relationship between ego-depletion—including cognitive, emotional, and behavioral depletion—and psychological ownership. This indicates that in high-trust environments, perceived institutional safeguards and social support networks provide psychological safety nets and resource compensation, thus attenuating depletion’s hindrance to psychological ownership development in new work roles. Furthermore, social trust positively moderated the direct effects of cognitive depletion and behavioral depletion on reemployment behavior. This implies that high-trust environments facilitate more positive attribution of cognitive biases, reduce threat perception of reemployment barriers, and provide alternative resources and behavioral cues, thereby directly buffering the impact of cognitive and behavioral depletion on reemployment.

However, no significant moderating effect of social trust was found between emotional depletion and reemployment behavior, potentially due to the multifaceted nature of emotional responses, where measurement challenges and complex modulation pathways by external factors may be implicated. The core reason why social trust cannot moderate the negative relationship between emotional depletion and reemployment behavior lies in the mismatch between the characteristics of emotional depletion and the operational mechanism of social trust. Emotional depletion is accompanied by profound psychological feelings such as tension and exhaustion, and it reflects the exhaustion of an individual’s emotional regulation resources. It exhibits strong implicitness and stability, and is rooted in deep-seated psychological issues—including separation from occupational roles after retirement and weakened self-identity—thus requiring the reconstruction of internal emotional experiences. In contrast, the moderating effect of social trust is mostly achieved by reducing external risk perception and providing a psychological safety net (e.g., alleviating doubts about competence at the cognitive level and restraining impulses at the behavioral level). Its role focuses on improving the uncertainty of the external environment, which makes it unable to address the needs for internal emotional recovery required to mitigate emotional depletion. Furthermore, the multifaceted nature of emotional responses complicates the regulatory pathways of external factors on emotional depletion. The existing operational mechanism of social trust fails to cover the recovery logic of emotional depletion, and therefore cannot buffer its inhibitory effect on reemployment behavior.

### 5.2 Policy recommendations

The reemployment of younger public sector retirees requires both accurate orientation and careful selection. It should not cause social conflict because of forced development, nor should it cause dissatisfaction among retirees because of excessive development. Based on the experience of developed countries in dealing with the reemployment of the younger elderly, and combined with the actual situation in China, the following countermeasures are proposed:

#### 5.2.1 Change backward concepts to reduce ego-depletion

As the leader in promoting active aging, the government should call on society and families to provide support, build an inclusive age-friendly environment, and focus on retirees’ psychological counseling to alleviate concerns and boost their reemployment initiative. Families need to reverse intergenerational support concepts to respond to retirees’ needs for social participation and reemployment. Communities should monitor local retirees’ situations and strengthen cohesion. Retirees themselves should maintain a positive attitude, break free from narrow family boundaries, and actively integrate into society.

#### 5.2.2 Safeguard the legitimate rights to enhance social trust

Judicial and relevant departments should rely on elderly associations and universities for the elderly to conduct legal popularization and education on elderly rights protection and supporting regulations, promoting standardized and regular legal publicity to make up for retirees’ lack of legal knowledge in rights protection and raise their law-abiding and rights-awareness. The government and judiciary should issue local regulations, clarify legal responsibilities, standardize supporting measures, fill legislative gaps in retirees’ protection, preferential treatment, education and services, and protect their rights from the legislative level.

#### 5.2.3 Build human resource information base to improve psychological ownership

In line with diversified social governance, human resources and social security departments should incorporate retired human resources into overall planning, establish and improve a retiree information database (classifying data by former professional expertise, age and skills), and build a vertical management network for retiree information to grasp their advantages and trends timely. Communities should set up information service stations for retirees, conduct surveys to clarify their numbers and situations, and use big data to build a community demand feedback platform and an online platform for reemployment intentions, providing convenient two-way information services.

#### 5.2.4 Build a lifelong education system to improve the comprehensive quality

As the Madrid International Plan of Action on Ageing states, “lifelong education is a prerequisite for elderly employment.” Universities for the elderly (retirees’ main knowledge channel) should add courses on law, finance and social welfare, offer special courses on social participation in public administration and education fields, organize practical activities to test learning outcomes, and hold salons or exhibitions to help capable retirees realize self-worth.

## 6 Research limitations and prospects

Although this study has achieved some research results, there are still some limitations. First of all, questionnaire was used to collect data, and the research results may be limited by social desirability, recall bias and other factors. And there is a certain degree of subjective cognitive bias. In the future, related research in this field can adopt a variety of research methods to improve the internal validity of the research. Secondly, this study only selected the young public sector retirees in four districts of Jinan City as the subjects. Therefore, it is necessary to conduct research on subjects with a wider and more uniform distribution in order to get a more representative and universal conclusion. At the same time, although the Western scale adopted in this paper has good validity, there may be deviations in the understanding of subjects due to the large gap between Chinese and Western cultures. Future research can develop measurement tools suitable for Chinese local culture to improve the validity of the scale in the Chinese context. Notably, a reverse causality may exist between ego-depletion and reemployment intention. Specifically, individuals with a stronger reemployment intention tend to exhibit a lower level of ego-depletion. Future studies should further validate the mutual relationship between these two variables, thereby enriching the body of research findings.

## Data Availability

The raw data supporting the conclusions of this article will be made available by the authors, without undue reservation.
